# Vaccination with Prion Peptide-Displaying Polyomavirus-Like Particles Prolongs Incubation Time in Scrapie-Infected Mice

**DOI:** 10.3390/v13050811

**Published:** 2021-04-30

**Authors:** Martin Eiden, Alma Gedvilaite, Fabienne Leidel, Rainer G. Ulrich, Martin H. Groschup

**Affiliations:** 1 Institute of Novel and Emerging Infectious Diseases, Friedrich-Loeffler-Institut, Federal Research Institute for Animal Health, Südufer 10, 17493 Greifswald-Insel Riems, Germany; fabienneleidel@icloud.com (F.L.); rainer.ulrich@fli.de (R.G.U.); martin.groschup@fli.de (M.H.G.); 2 Life Sciences Center, Institute of Biotechnology, Vilnius University, Saulėtekio al. 7, LT-10257 Vilnius, Lithuania; alma.gedvilaite@bti.vu.lt; 3 Task Force Animal Diseases, Darmstadt Regional Administrative Council, Luisenplatz 2, 64283 Darmstadt, Germany

**Keywords:** prion disease, polyomavirus, virus-like particles, active immunisation

## Abstract

Prion diseases like scrapie in sheep, bovine spongiform encephalopathy (BSE) in cattle or Creutzfeldt–Jakob disease (CJD) in humans are fatal neurodegenerative diseases characterized by the conformational conversion of the normal, mainly α-helical cellular prion protein (PrP^C^) into the abnormal β-sheet rich infectious isoform PrP^Sc^. Various therapeutic or prophylactic approaches have been conducted, but no approved therapeutic treatment is available so far. Immunisation against prions is hampered by the self-tolerance to PrP^C^ in mammalian species. One strategy to avoid this tolerance is presenting PrP variants in virus-like particles (VLPs). Therefore, we vaccinated C57/BL6 mice with nine prion peptide variants presented by hamster polyomavirus capsid protein VP1/VP2-derived VLPs. Mice were subsequently challenged intraperitoneally with the murine RML prion strain. Importantly, one group exhibited significantly increased mean survival time of 240 days post-inoculation compared with 202 days of the control group. These data show that immunisation with VLPs presenting PrP peptides may represent a promising strategy for an effective vaccination against transmissible spongiform encephalitis agents.

## 1. Introduction

Transmissible spongiform encephalopathies (TSE) are fatal neurodegenerative disorders that are caused by proteinaceous infectious particles (prions). So-called prion diseases include Creutzfeldt–Jakob Disease (CJD) and Kuru in humans, bovine spongiform encephalopathy (BSE) in cattle, scrapie in sheep and goats, and chronic wasting disease (CWD) in cervids [1].

Prion diseases are caused by the misfolding of the normal, host-encoded cellular prion protein (PrP^C^) into a pathogenic isoform (PrP^Sc^). Cellular PrP^C^ is linked to the cell membrane by a glycophosphatidyl inositol (GPI) anchor and contains two N-linked carbohydrate groups, and a single disulfide bond [1,2]. PrP^C^ is found in all mammals, and most highly expressed in the central nervous system, but also in a vast majority of immune cells like lymphocytes, natural killer cells and monocytes. Moreover follicular dendritic cells (FDC) are essential sites of prion replication in lymphoid tissues [3]. The exact biological function of PrP^C^ is still unknown but there are indications for a role in signal transduction [4], immunoregulation [5], synaptic plasticity through promoting neurite outgrowth [6], Cu^2+^ binding [7,8] and modulating N-methyl-D-aspartate receptor (NMDAR) activity [9]. The potential neuroprotective function of PrP^C^ is consequently lost during its conversion to PrP^Sc^ disturbing synaptic functionality and leading finally to synapse degeneration [10]. 

During the conversion process, α-helical and coiled structures of PrP^C^ are refolded into β-sheets inducing aggregation into fibrils and formation of β-sheet-rich amyloids. This structural transition causes changes of the biochemical properties of the prion protein including partial resistance to proteases like proteinase K, aggregation and fibril formation [1]. 

Key target for therapies against prion disease is the prion protein and enormous efforts have been undertaken to identify factors that interfere with its conversion and/or aggregation. A large number of anti-prion compounds have been identified so far: amyloid binding dyes like Congo red [11] and sulfated polyanions like pentose polysulfate [12], antibiotics e.g., amphotericin B [13], natural polyphenols like curcumin, tannic acid or green tea-derived epigallocatechin gallate [14] as well as tea from *Scutellaria* herb [15]. In addition, various chemical compounds with in vitro anti-prion activity like tetrapyrroles [16] or piperazines [17,18] were described. Many of them were tested for therapeutic approaches and several prolonged incubation times, but without curative effect. One disadvantage of many of these compounds is their inability to cross the blood-brain barrier. A source of anti-prion compounds with a high blood–brain barrier permeability were approved drugs like quinacrine with ambiguous effects in scrapie agent-infected mice [19]. In addition, quinacrine was tested in human CJD patients, again with no or only marginal effects. Flupirtine was tested in a clinical trial in CJD patients and displayed mild positive effects on cognitive functions, but no prolongation of survival times. Finally, amantadine, pentosan polysulfate and doxycycline were applied in other clinical trials also showing no benefits [20]. 

An alternative approach represents immunotherapy either by passive or active immunisation. Passive immunisation by intraperitonial application of antibodies that inhibit PrP conversion lead to prolonged incubation periods in infected mice [21,22] or even kept the mice completely healthy for more than 300 days [23]. However, these antibodies were unable to cross the blood–brain barrier thus preventing therapeutic efficacy on disease progression in the central nervous system. In addition, many therapeutically effective anti-PrP antibodies exhibit neurotoxic properties in brain tissues as well [24].

The induction of a prion-specific antibody response following active immunisation is hampered by self-tolerance phenomena to the host encoded endogenous PrP^C^. Nevertheless, antibody responses against PrP can be induced by (a) modified peptides representing either truncated or cross-linked variants or dimers, (b) DNA vaccines encoding PrP specific sequences or (c) by bacterial or viral vectors that overcome the immune tolerance. However, the therapeutic or preventive effects in animal models are limited so far [25,26]. 

Virus-like particles (VLPs) can be generated by the spontaneous assembly of heterologously expressed viral capsid and/or envelope proteins [27]. The major capsid protein VP1 of hamster polyomavirus (HaPyV; *Mesocricetus auratus polyomavirus 1*) assembles to VLPs upon expression in yeast *Saccharomyces cerevisiae* and tolerates the insertion of foreign protein segments at selected surface-exposed sites in chimeric VLPs [28,29], for review see [30]. The insertion capacity of the HaPyV-derived VLPs allows the presentation of foreign peptides of 120 amino acid (aa) length, but can be enhanced by the production of pseudotype VLPs consisting of unmodified VP1 carrier and VP2 fusion protein with foreign insertions [21,22,23,24,25,26,27,28,29,30,31,32,33,34,35,36]. In addition, the introduction of a flexible glycine-serine-serine-glycine (GSSG) linker in VP1 improved the carrier [32,37]. Chimeric VP1-derived VLPs are highly immunogenic, even without adjuvant co-application, and can induce strong and protective immune responses [32,37,38]. Chimeric VLPs were successfully applied for the generation of insert-specific monoclonal antibodies [32,33].

In this work, we studied whether it is possible to induce PrP-specific antibody responses in wild type mice and whether these have protective effects after challenge with scrapie prions. To address this issue, full length as well as partial murine PrP sequences were inserted at different positions of the viral capsid proteins VP1 or VP2 of HaPyV. The generated chimeric and pseudotype VLPs were applied to wild-type C57/Bl6 mice that were subsequently inoculated with mouse scrapie strain RML and assayed for protective effects on prion disease progression. 

## 2. Materials and Methods

### 2.1. Antibodies and Prion Protein

Monoclonal antibody (mAb) 3F4 was kindly provided by M. Beekes (Robert Koch-Institute, Berlin, Germany), mAb SAF70 was received from Tecan (Männedorf, Switzerland) and mAb 8H4 from Sigma (Darmstadt, Germany). Bacterial expressed recombinant murine prion protein (MPrP, Uniprot Nr: P04925) was obtained from abcam (Berlin, Germany).

### 2.2. Construction of Expression Plasmids

Enzymes and kits for DNA manipulations were purchased from Thermo Fisher Scientific Baltics (Vilnius, Lithuania). All DNA manipulations and construction of plasmids were carried out according to standard procedures and manufacturers recommendations [39]. Recombinants were screened in *Escherichia coli* K12 DH5α. HaPyV VP1 genes with modified VP1 coding region for insertion of sequences encoding peptides of interest into either position #1 (corresponding to amino acids 80–89) or position #4 with GSSG linker (corresponding to amino acids 288–295) were described previously [29,37]. Vector pFGG3-VP1/VP2Bg was used for fusing MPrP peptide encoding sequences to VP2 gene as described previously [35]. Five PrP fragments encoding peptides of different sizes: 37 amino acids: PrP1 (K1, K9), 46 amino acids: PrP2 (K6), 92 amino acids: PrP3 (K7, K8), 78 amino acids: PrP4 (K2, K4, K5) and 209 amino acids: MPrP(K3) (see Table 1) were amplified by PCR from a 3f4-tagged murine prion gene that codes for a murine PrP with a tetrapeptide Met-Lys-His-Met at positions 109–112 (3F4 epitope). Primers with the following sequences were used: 23Dir: 5′gcggatcctaaaaagcggccaaagcctggaggg3′; 51Dir: 5′ gcggatcctcagggtggcacctgggggcagc3′; 128Dir: 5′ gcggatcctatgctggggagcgccatgagcagg3′; 174Dir: 5′ gcggatccattcgtgcacgactgcgtca atatc3′; 128Rev: 5′ gcggatccatgtagccaccaaggccccccactac3′; 164Rev: 5′ gcagatctggcctgtagt acacttggttaggg3′; 219Rev: 5′ gcggatccttctggtactgggtgacgcacatc3′; 231Rev: 5′ gcggatccgagct ggatcttctcccgtcgtaatagg3′. 

PCR amplified PrP1, PrP2, PrP3, PrP4 and MPrP peptide-encoding DNA fragments with flanking BamHI sites for cloning were inserted into pTZ57 vector (Thermo Fisher (Fermentas)) and verified by DNA sequencing. BamHI digested DNA fragments encoding PrP1, PrP2, PrP3, and PrP4 peptides were inserted into the BglII site at insertion sites #1 and #4 of VP1-encoding gene in the pFX7 vector [28] and DNA fragments encoding PrP3, PrP4 and MPrP peptides were inserted into BglII site in pFGG3-VP1/VP2Bg vector [35] for fusion with modified HaPyV VP2-encoding gene. The constructed recombinant plasmids for the expression of chimeric and pseudotype VLPs presenting PrP peptides are listed in Table 1. 

### 2.3. Generation, Purification and Electron Microscopy Analysis of Chimeric and Pseudotype Virus-Like Particles (VLPs)

The chimeric VP1 and VP2 proteins of HaPyV were produced in yeast *S. cerevisiae* strain AH22 -214 (a, leu2 his4). Purification of recombinant proteins was performed as described previously [35,40]. Briefly, yeast cells transformed with constructed plasmids (Table 1) were first cultured in glucose-, then galactose-containing induction media for 24 and 18 h, respectively and then collected by centrifugation, washed with distillate water, and stored at −20 °C until purification. For purification of chimeric proteins, yeast cells after suspension in DB450 buffer (450 mM NaCl, 1 mM CaCl_2_, 0.001% 0.25 M L-arginine, Triton X-100, 10 mM Tris/HCl, pH 7.2) with 2 mM phenylmethylsulfonylfluorid (PMSF) and EDTA-free Complete Protease Inhibitor Cocktail tablets (Roche Diagnostics, Mannheim, Germany) were disrupted with glass beads using Bead-Beater GB26 (BioSpec Products, Inc., Bartlesville, OK, USA). The chimeric proteins were purified from the supernatant of yeast cell lysate by ultracentrifugation overnight at 4 °C in 20–60% sucrose gradient (100,000× *g*) and then for 18 h in cesium chloride (CsCl) gradient (1.23–1.38 g/mL). After pooling and diluting, purified recombinant protein-containing fractions in DB150 buffer were precipitated by ultracentrifugation for 4 h at 100,000× *g*. Pellets containing recombinant chimeric proteins were dissolved in phosphate-buffered saline (PBS, pH 7.2), dialyzed against PBS, aliquoted (25 and 50 μg), lyophilised and stored until use. 

To confirm the VLP assembly of purified recombinant proteins, samples of each construct were placed on 300-mesh carbon-coated palladium grid. Adhered to the grid, samples were negatively stained with 2% aqueous uranyl acetate solution and examined by JEM-100S electron microscope (JEOL, Tokyo, Japan). 

### 2.4. Sodium Dodecyl Sulfate Polyacrylamide Gel Electrophoresis (SDS-PAGE) and Western Blot Analysis 

Samples of yeast lysates or purified recombinant proteins were mixed with the sodium dodecylsulfate (SDS)- polyacrylamide gel (PAGE) sample buffer (Thermo Fisher Scientific Baltics, Vilnius, Lithuania), boiled for 5 min, applied to a 12% SDS-PAGE mini gel and run in SDS-Tris-glycine buffer. After fractionation proteins were stained with Coomassie brilliant blue (Sigma–Aldrich, St. Louis, MO, USA) for protein band visualization or transferred to polyvinyldifluoride (PVDF) membranes (Merck-Millipore) under semi-dry conditions for immunoblot analysis, as previously described [40]. Briefly, the membranes were blocked with 5% milk in TTBS (0.1 M Tris, 0.3 M NaCl, pH 7.4 and 0.1% Tween 20) for 2 h (h), rinsed with TTBS and incubated overnight with primary antibody at room temperature (RT). For detection of HaPyV VP1-derived chimeric proteins monoclonal antibody 6D11 [32] (1:1000 dilution) and for detection of HaPyV VP2 protein an in-house mouse polyclonal antiserum raised against HaPyV VP2 protein (1:500 dilution) were used. After rinsing in TTBS, the membranes were incubated with horse radish peroxidase (HRP)-labeled anti-mouse IgG (Bio-RadLaboratories, 1:2000) for 1 h at room temperature (RT). After several rinsing steps the enzymatic reaction was developed using 4-chloro-1-naphtol (BioChemica, Darmstadt, Germany) and hydrogen peroxide (Carl Roth GmbH, Karlsruhe, Germany) in TBS (0.1 M Tris, 0.3 M NaCl, pH 7.4) or TMB-blotting ready-to-use substrate (Sigma-Aldrich). Prestained protein molecular weight (MW) markers were purchased from Thermo Fisher Scientific Baltics.

Accumulated PrP^Sc^ was examined by a western blotting protocol using phosphotungstic acid (PTA) precipitation [41,42]. In short, 10% (*wt*/*vol*) brain homogenates of all samples were homogenized in 0.42 mM sucrose solution containing detergents (0.5% deoxycholic acid sodium salt/0.5% Nonidet P-40) and incubated with proteinase K (PK, final concentration: 50 µg/mL) at 55 °C for 60 min. Subsequently the digestion was stopped by addition of Pefabloc (Roche, Mannheim, Germany) and heating at 95 °C for 5 min. Subsequently, PTA was added to the samples (final concentration, 0.3%), incubated at 37 °C for 60 min and centrifuged at 13,300 rpm for 30 min at RT. Samples were resuspended in loading buffer, heated for 5 min at 95 °C and loaded on 16% Tris-PAGE. Proteins were transferred onto PVDF membrane (Millipore) and the membranes were blocked for 1 h in PBST-milk. PrP^Sc^ was detected using mAb SAF70 (Bertin Technologies, Montigny le Bretonneux, France) by incubating the membranes for 1 h at RT. After washing the membranes 3 times with PBST a secondary antibody—alkaline-phosphatase-conjugated anti-mouse immunoglobulin (Dianova, hamburg, Germany)—was added in PBST for 1 h at RT. The membranes were finally washed 3 times in PBST and bound antibodies were detected with the chemiluminescence substrate CDP Star (Thermo Fisher) and signals were visualized and calculated with Versa Doc system (Quantity One, Bio-Rad, Munich, Germany) and by the analysis software Quantity One (BioRad, Munich, Germany) using background substraction method.

### 2.5. Propagation of Scrapie Strain and Immunisation Scheme

**Figure 1:**
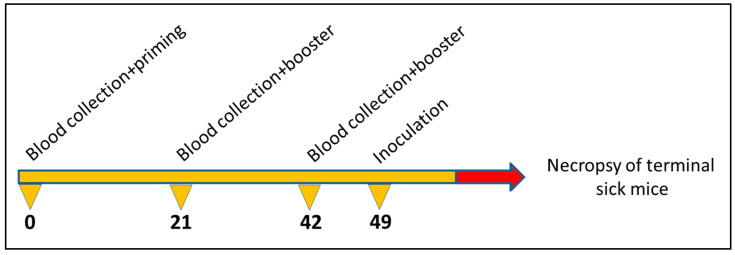
Immunisation and sample collection regimen with indication of treatment dates (days).

Ten groups of C57/Bl6 mice (5 individuals each) were subcutaneously (s.c.) immunized in a prime-boost regimen three times at intervals of three weeks (priming, first and second boost) with 50 µg of the corresponding chimeric or pseudotype VLPs or VP1/VP2-derived VLPs (control). Immunized mice were subsequently challenged with 50 µL of a 1% brain homogenate of mouse scrapie strain RML intraperitoneally (i.p.). The schematic course of application is depicted in Figure 1. The used mouse scrapie strain RML was originally designated the “Chandler” strain and was the result of a passage of natural sheep scrapie brain to mice [43]. The health status of the mice was analysed daily, and body weight was recorded weekly. After the onset of TSE-associated clinical symptoms including weight loss, abnormal tail tonus, hind limb paralysis, the animals were euthanized. The incubation times were calculated as the time between inoculation and necropsy. The brains were removed and stored at −20 °C. Mice that died from other causes than scrapie were excluded from the dataset. Animal experiments were approved by German law (reference number: LALLF M-V/TSD/7221.3-1.1-039/05). 

### 2.6. Enzyme-Linked Immunosorbent Assay (ELISA)

All serum samples were tested in an in-house indirect IgG enzyme-linked immunosorbent assay (ELISA) based on full length recombinant murine PrP and HaPyV VP1 protein. Maxisorb immunoplates (Nunc, Roskilde, Denmark) were coated with 1 µg of the corresponding protein, diluted in 0.05 M carbonate–bicarbonate buffer (pH 9.6). After incubating the plates overnight at 4 °C, they were washed three times with 300 µL washing buffer PBST, followed by a blocking step with 200 µL/well 10% skim milk powder diluted in PBS for 1 h at 37 °C. Serum (5 µL diluted in 120 µL 2% skimmed milk) was added, plates were incubated at 37 °C for 1 h and subsequently washed. 100 µL of HRP-conjugated protein G (Calbiochem), diluted 1:5000 in dilution buffer, were then added to each well. After another 1 h incubation step, plates were washed again and 100 µL substrate (2′-Azinobis [3-ethylbenzothiazoline-6-sulfonic acid]-diammonium salt (ABTS); Roche, Mannheim, Germany) was added to each well. Reactions were run for 30 min at RT in the dark, stopped by addition of 1% SDS and the optical density (OD) value at 405 nm was determined. Relative OD was calculated as OD from samples at corresponding timepoints t_0_, t_21_ and t_42_ subtracted from t_0_ value and divided by t_0_ [(OD_tx_ − OD _t0_)/OD_t0_.]

### 2.7. Statistical Analysis

Survival times were analyzed by Kaplan–Meier survival analysis using the log-rank test to compare the curves. The statistical analysis was done using SigmaBlot statistical software (Systat Software, San Jose, CA, USA). Survival times are expressed as means ± standard deviation.

## 3. Results

### 3.1. Biochemical and Electron-Microscopical Characterization of VLPs

**Figure 2:**
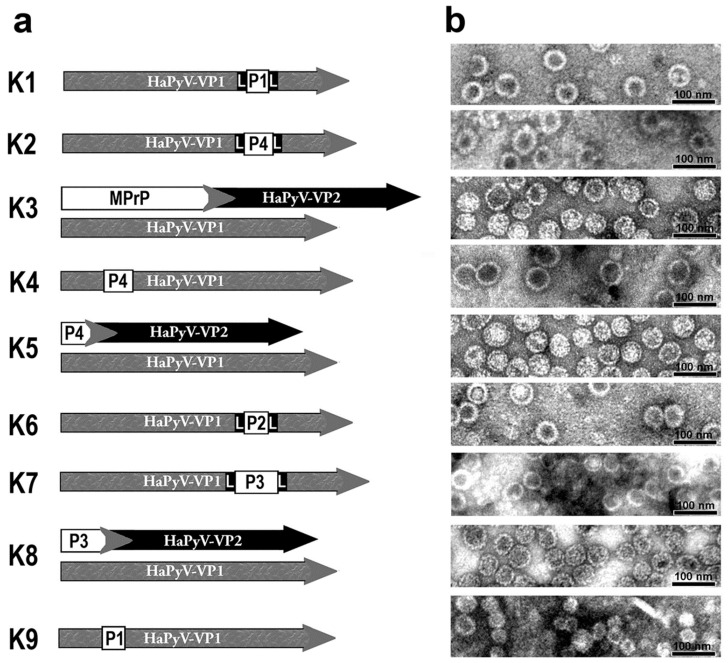
Schematic illustration of hamster polyomavirus (HaPyV) VP1 or VP2 fusion proteins with murine prion protein (MPrP) peptides (**a**) and detection of formation of virus-like particles by negative staining electron microscopy (**b**). L, linker GSSG.

Partial sequences of MPrP were inserted into major capsid protein VP1 or fused at N-terminus of a N-terminally truncated capsid protein VP2 of HaPyV. This included MPrP amino acid residues 128–164 (P1), 174–219 (P2), 128–219 (P3), 51–128 (P4) as well as the full length mature MPrP sequence (residues 23–231, MPrP). The structure of the nine different VP1 or VP2 fusion proteins is schematically illustrated in Figure 2a. All constructs assembled into polyomavirus-derived VLPs are as shown by electron microscopy (Figure 2b).

**Figure 3:**
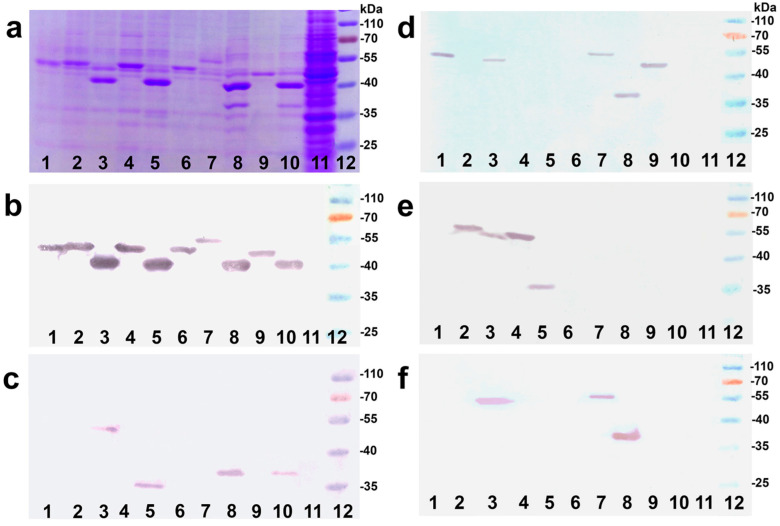
Analyses of purified proteins expressed by constructs K1 to K9 (lanes 1–9), VP1/VP2 protein controls (lane 10) and of lysate of non-transformed yeast cells (lane 11) by sodium dodecyl sulfate polyacrylamide gel electrophoresis (SDS-PAGE) (**a**) and Western blot assay with VP1 specific (**b**), VP2 specific (**c**) and PrP specific antibodies SAF70 (**d**), 3F4 (**e**) and 8H4 (**f**). Pre-stained marker (lane 12) encompassing 25–70 kDa.

The constructs were detected by Coomassie blue staining (Figure 3a) as well as in Western blot assay using PrP specific as well as VP1/VP2 specific antibodies (Figure 3b–f). All VLPs displayed immunoreactivity against VP1-specific mAb 6D11 (Figure 3b) and all pseudotype VP1/VP2 VLPs (K3, K5, K8) in addition against polyclonal anti-VP2 antiserum (Figure 3c). Detection of MPrP peptides was undertaken with mAb SAF70 (Figure 3d), mAb 3F4 (Figure 3e) and mAb 8H4 (Figure 3f), targeting amino acid residues 142–160, 109–115, 145–180, respectively. The detected protein bands corresponded to the predicted molecular mass of constructed chimeric proteins (see Table 1).

### 3.2. Serological Analysis of VLP-Immunized Mice 

**Figure 4:**
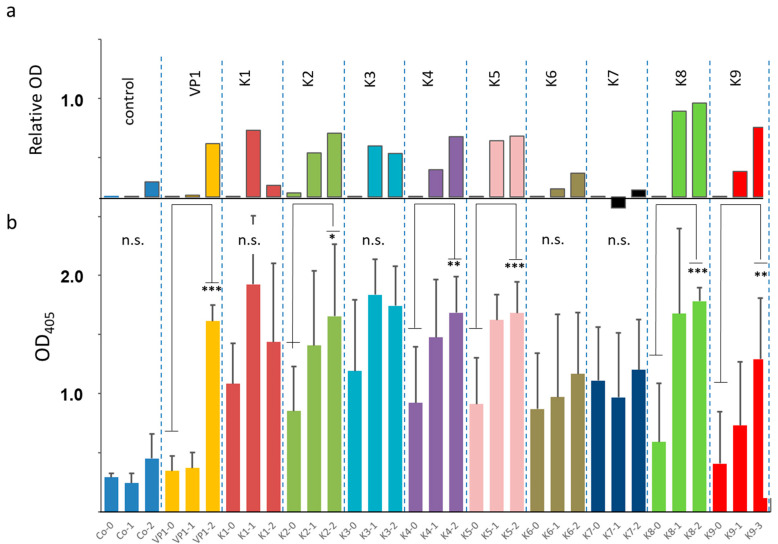
Optical density (OD) of indirect VP1 IgG enzyme-linked immunosorbent assay (ELISA) displaying relative OD (**a**) as well as raw data (**b**) from mice sera after immunisation with corresponding VLP constructs K1–K9 at three different timepoints: 0 (priming), 1 (21 days, first boost) and 2 (42 days, second boost). Indirect ELISA is performed with yeast-expressed VP1-protein as antigen. Controls include non-immunized (Co) and VP1/2 immunized mice. * (*p* < 0.05); ** (*p* < 0.005); *** (*p* < 0.001).

To assess, whether antibodies were induced by the immunisation procedure, sera at time point 0 (priming), day 21 (first boost) and day 42 (second boost) were collected and analysed by an indirect ELISA using homologous constructs as antigens (Figure 4). The individual data are shown in Table S1. There was no correlation between individual ELISA values and incubation times of corresponding mice (Table S2).

**Figure 5:**
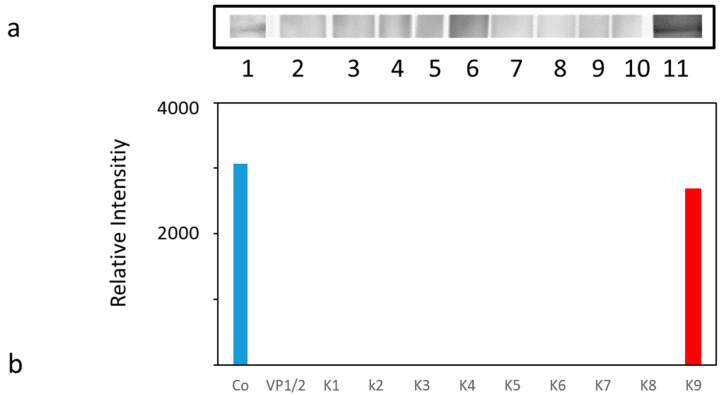
(**a**) Western blot analysis of pooled sera from animals immunized with VP1/2 control (VP1, lane 2), K1 (lane 3), K2 (lane 4), K3 (lane 5), K4 (Lane 6), K5 (lane 7), K6 (lane 8), K7 (lane 9), K8 (lane 10), K9 (lane 11). Each lane contains blotted recombinant murine prion protein separated on an 16% SDS-PAGE and probed with corresponding serum pools. mAb SAF70 (lane 1) was used as a positive control (Co). (**b**) Semi-quantitative analysis by VersaDoc. Western blot signals were measured with a digital camera and evaluated with the respective software (Quantity One).

Five constructs (K2, K4, K5, K8 and K9) including the VP1/2 control developed a significant increase in the anti-VP1 antibody response against the applied VLP construct. Four constructs (K1, K3, K6 and K7) did not provoke a significant induction of anti-VP1 antibody response. No PrP specific antibodies were detected in ELISAs on days 21 and 42, when full length recombinant MPrP was used as immunogen). To reveal low antibody levels mouse sera taken at necropsies were pooled (100 µL) (from each group of 5 animals) and used in Western blot tests (final dilution 1:20). A specific signal was obtained only for pooled sera from the K9 immunized animals. No signals were seen for any other immunogen and none for the VP1/2 control (Figure 5a). The semi-quantitative analysis is shown in Figure 5b. The corresponding Western blot test is depicted in Figure S1. 

### 3.3. In Vivo Efficacy of VLP Immunisation against Scrapie Infection

**Figure 6:**
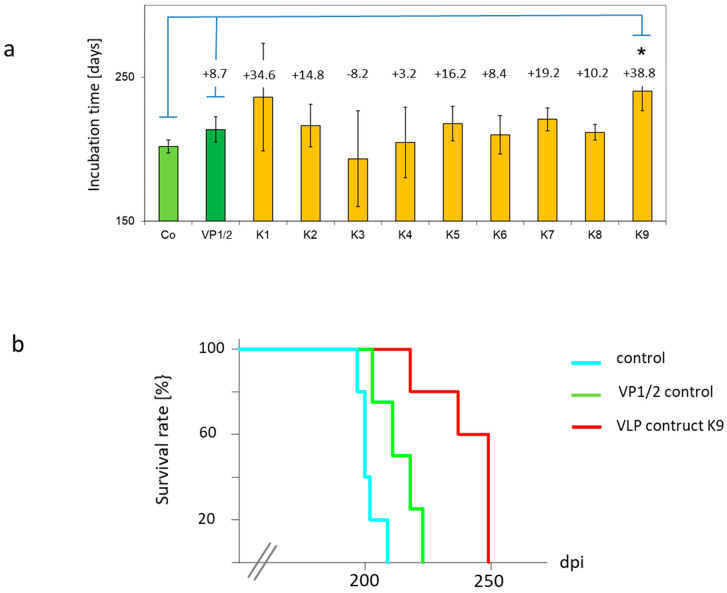
VLP construct K9 prolongs incubation time in mice challenged with murine RML scrapie prions. Comparison of mean survival times ± the standard deviation for mice immunized with VLP constructs K1-K9, VP1/2 control and non-immunized control (Co) mice (**a**). Columns are labeled with elongation time [days] compared to untreated control. Kaplan–Meier survival analysis of intraperitoneally (i.p.) infected mice after s.c. treatment with construct K9. Controls included untreated controls (Co) and VP1/2 controls (**b**).

The protective potential of all VLP constructs was measured by determination of the mean survival time after challenge (Figure 6). Untreated mice had a mean incubation time of 201.6 +/− 4.5 days. Mice treated with the VP1/2 control alone exhibited an incubation time of 213.8 +/− 8.7 days. A slight increase was observed in mice groups treated with almost all constructs ranging from 204.8 +/− 24.5 days (construct K4) up to 236.3 +/− 37.3 days (construct K1). Treatment with construct K3, which encompasses the whole PrP sequence, indicated a reduced survival time of 193.4 +/− 33.23 days. In contrast, immunisation with construct K9 led to a significant prolongation of incubation time of about 40 days (240.4 +/− 13.6 days versus 201.6 +/− 4.5 days). Individual data of each animal are depicted in Table S2.

**Figure 7:**
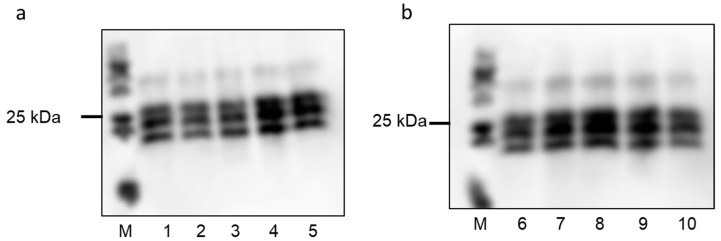
PrP^Sc^ accumulation in the brains of selected mice challenged with RML. Immunoblot analysis of RML-infected mouse brain homogenate digested with proteinase K (PK). (**a**) PrP^Sc^ of scrapie infected mice without VLP treatment and (**b**) PrP^Sc^ of scrapie infected mice immunized with VLP construct K9. Detection of PrP^Sc^ was carried out with mAb SAF70. (M) molecular weight marker.

The PrP^Sc^ accumulation in brains of mice immunized with construct K9 and untreated terminally sick mice was analyzed by Western blot test (Figure 7). 

Western blot analysis of PK resistant PrP^Sc^ fragments demonstrated no differences in the glycosylation pattern nor molecular weight of PrP^Sc^ between VLP (K9) immunized and control mice. The analysis demonstrates further that the amount of PrP^Sc^ accumulation was similar in control compared to immunized mice. Similar findings were observed for mice immunized with the other constructs (Figure S2).

## 4. Discussion

The results demonstrate that HaPyV capsid protein-derived VLPs containing PrP fragments represent a promising prophylactic strategy for direct immunisation. In total, nine VLP variants displaying different PrP fragments were tested and one construct (K9)—encompassing PrP sequence 128–164—significantly elongated the survival time. Mice immunized with this construct K9 exhibited a humoral immune response and generated auto-antibodies that detect PrP as determined by western blot assay. 

Interestingly, construct K1 chimeric VLPs harboring the same peptide P1 (amino acids 128–164) also showed, although it was not significant, a prolongation of the incubation period (+34.6 versus +38.8 for K9). The different potential of K9 and K1 chimeric VLPs might be explained by the presence/absence of the GSSG linker or the different insertion sites (site #1 in K9 versus site #4 in K1). The lacking or much lower effect of pseudotype-based VLPs might be explained by the insertion of other peptides; the lower PrP epitope density on the VLP surface might be an additional reason to be tested in further experiments with P1 peptide fused to VP2 protein of HaPyV. Moreover, a slight, but non-significant increase in the incubation time (12 days) was generally observed only for pure VP1/VP2 derived VLPs. VLPs have been shown to trigger a cellular immune response including the release of pro-inflammatory cytokines and a cytotoxic T-lymphocyte (CTL) response [44]. This stimulation activates macrophages to phagocytosis leading to a partial degradation within the phagolysosomal compartments of the cells [45].

In a previous study, direct intracerebral inoculation of mouse scrapie strain RML lead to an incubation time of 164 days in C57BL/6 mice [46]. In contrast intraperitoneal inoculation of this strain in our study prolonged incubation time by 38 days due to initial dissemination of PrP^Sc^ via peripheral nerves and lymphoreticular organs to the CNS followed by brain neuroinvasion [47]. Application of construct K9 exhibits a similar delay of 38 days (240 vs. 202 days) and underlines the significant immunisation effect of these chimeric VLPs.

The use of VLPs comprising PrP sequences to elicit an immune response against PrP have been evaluated in just a few studies: in one approach a peptide of nine amino acids of the murine PrP (DWEDRYYRE; amino acids 144–152) was inserted within the L1 major capsid protein of bovine papillomavirus type 1 (BPV-1). The assembled VLPs induced antibodies in rabbits, that inhibited the synthesis of PrP^res^ in prion-infected ScN2a neuroblastoma cells [48]. In an alternative setting murine leukemia virus (MLV) was used to create VLPs displaying murine PrP that induced anti-PrP^C^ specific antibodies even in wild-type mice [49]. Interestingly, this was only observed for constructs encoding the C-terminal prion fragment (amino acids 121 to 231). However, in both cases, no additional studies have been performed so far to further evaluate these findings in prophylactic and therapeutic settings. 

The elongation of the incubation time of about 18% (240 vs 202 days) observed here is in a similar range compared to other immunisation strategies: immunisation with murine PrP linked to Keyhole Limpet Hemocyanin (KLH) prior to infection prolonged incubation time by 11.2% for scrapie in mice [50] and scrapie infected mice that were vaccinated with aggregated murine PrP (suspended in complete Freund’s adjuvant) survived approximately 28 days longer than naive mice [51]. Immunisation with KLH-linked hamster PrP peptides resulted in a prolongation of about 13.0–18.7% in scrapie infected hamsters [52] and the immunisation of hamsters with dendritic cells loaded with PrP peptides (encompassing amino acids 98–127) delayed the onset of clinical scrapie by 18.7% [53].

Another promising approach to overcome PrP self-tolerance was the immunisation with recombinant dimeric murine PrP with different adjuvants which induced in vitro efficacious anti-PrP antibodies [54,55]. A similar approach with monomeric and dimeric PrP induced auto-antibodies against cervid PrP and significantly prolonged incubation times in CWD-infected transgenic elk PrP (TgElk) mice [56,57]. Interestingly, immunisation with heterologous monomeric mouse PrP caused the largest prolongation by 60% in TgElk mice [57].

The exact mechanism that causes the significant delay remains to be elucidated, but the generated antibodies could interfere with the binding and/or conversion into the infectious PrP^Sc^ isoform, since the expressed PrP sequence in construct K9 encompasses a specific region of the globular domain (amino acids 128–164). This includes an α-helix located at amino acid positions 143 to 153 and two short β-sheets (residues 127–130 and 160–163). The first helix plays a significant role in binding cellular PrP^C^ to infectious PrP^Sc^ and the two β-sheets are initial point of the conformational transition into a β-solenoid architecture and subsequent PrP^Sc^ amyloid fibril formation [58]. Antibodies targeting this region have been demonstrated to have a significant anti-PrP effect, e.g., mAb 6H4 (raised against amino acids 144–152) which clears PrP^Sc^ from prion infected neuroblastoma cells [59]. Moreover, passive immunisation with mAb ICSM18 (targeting amino acid residues 146–159) lead to a prolongation of the incubation period by more than 500 days [23]. Therefore, the VLP-induced antibodies could act as inhibitors of PrP conversion. On a molecular level, antibodies could interfere directly with host encoded PrP^C^ to avoid PrP^Sc^ binding and to prevent subsequent conversion into pathogenic isoform due to lack of substrate. Alternatively antibodies could directly bind to cellular PrP^C^ and trigger a decrease of PrP^C^ levels in host cells which are then not available as a source for further conversion and subsequent PrP^Sc^ replication. This mechanism has been proposed for mAb targeting PrP residues 144–152, which causes clearance of cellular PrP in prion-infected cell cultures [60]. However, the proposed inhibitory mechanisms are only transient, as terminal sick mice, either VLP treated or not, develop a similar PrP^Sc^ aggregate pattern and do not effectively cure the disease.

## 5. Conclusions

Taken together, the obtained results demonstrate that a homologous chimeric VLP-based PrP vaccine can abrogate host encoded immune tolerance to cellular PrP^C^ and induces an albeit weak antibody response that prolongates survival time in scrapie infected mice. Further studies are needed to compare the chimeric to the pseudotype PrP VLP vaccine and determine the potential of a PrP-VLP vaccine for prophylactic or even therapeutic interventions in currently fatal and incurable prion diseases.

## Figures and Tables

**Table 1:** List of constructed recombinant plasmids for the expression of chimeric and pseudotype virus-like particles (VLPs) presenting prion protein (PrP) peptides.

Abbreviation	Plasmid	Expressed Protein	PrP Amino Acid Sequence *	Predicted Molecular Mass of Expressed Proteins [kDa]
K1	pFX7-VP1-PrP1-4L	VP1-PrP1-4L	128-164	47.5
K2	pFX7-VP1-PrP4-4L	VP1-PrP4-4L	51-128	50.5
K3	pFGG3-VP1/VP2-MPrP	VP1 and VP2-MPrP	23-231	42.55 and 52.5
K4	pFX7-VP1-PrP4-1	VP1-PrP4-1	51-128	49.2
K5	pFGG3-VP1/VP2-PrP4	VP1 and VP2-PrP4	51-128	42.5 and 37.0
K6	pFX7-VP1-PrP2-4L	VP1-PrP2-4L	174-219	48.2
K7	pFX7-VP1-PrP3-4L	VP1-PrP3-4L	128-219	64
K8	pFGG3-VP1/VP2-PrP3	VP1 and VP2-PrP3	128-219	42.5 and 40.5
K9	pFX7-VP1-PrP1-1	VP1-PrP1-1	128-164	46.3
control	pFGG3-VP1/VP2	VP1 and VP2	-	42.5 and 38.8

* numbering according to Uniprot Nr: P04925 (PRIO_MOUSE), L—glycine-serine-serine-glycine (GSSG) linker.

## Data Availability

All data of this study are available within this manuscript and its Supplementary Materials.

## Supplementary Materials

The following are available online at https://www.mdpi.com/article/10.3390/v13050811/s1, Table S1: Optical density (OD) of indirect VP1 IgG ELISA displaying raw data from mice sera after immunisation with corresponding VLP constructs at three different timepoints, Table S2: Survival time (individual values) of RML-infected C57/Bl6 mice immunized with virus-like particles, Figure S1: Western blot analysis Western blot analysis of pooled sera from immunized animals, Figure S2: PrP^sc^ accumulation in the brains of selected mice challenged with RML.
