# Supplementary material for: Vaccination with Prion Peptide-Displaying Polyomavirus-Like Particles Prolongs Incubation Time in Scrapie-Infected Mice
Source: Viruses. 2021 Apr 30;13(5):811. doi: 10.3390/v13050811 (PMC8147134; doi:10.3390/v13050811)
Supplement: Supplementary file 1 [file viruses-13-00811-s001.zip › viruses-1182781-supplementary.pdf]

Table S1: Optical density (OD) of indirect VP1 IgG ELISA displaying raw data from mice sera after immunisation with corresponding VLP constructs at three different timepoints: 0 (priming), 1 (21 days, first boost) and 2 (42 days. second boost). Indirect ELISA is performed with yeast-expressed VP1-protein as antigen. Controls include non-immunised (Co) and VP1/2 immunised mice

**OD405**	**animal 1**	**animal 2**	**animal 3**	**animal 4**	**animal 5**
Co-0	0.32	0.26	0.31	0.24	0.30
Co-1	0.41	0.20	0.22	0.22	0.19
Co-2	0.39	0.70	0.68	0.48	0.22
VP1/2-0	0.32	0.40	0.51	0.32	*
VP1/2-1	0.27	0.28	0.37	0.34	*
VP1/2-2	1.44	1.70	1.63	1.60	*
K1-0	1.22	1.60	1.06	1.22	1.19
K1-1	2.05	2.32	0.65	1.92	2.07
K1-2	0.72	2.10	0.54	1.69	1.49
K2-0	1.68	0.56	0.72	0.60	0.84
K2-1	1.94	0.52	0.88	2.11	1.96
K2-2	2.22	2.27	1.15	1.82	1.87
K3-0	0.33	0.70	1.10	0.91	1.55
K3-1	2.33	1.55	1.51	1.98	2.07
K3-2	2.24	1.37	1.67	1.67	1.99
K4-0	0.78	0.47	0.46	0.50	1.50
K4-1	1.31	1.27	2.08	1.21	2.08
K4-2	1.82	1.24	2.18	1.79	1.77
K5-0	0.88	0.46	0.46	0.76	*
K5-1	2.09	1.50	1.60	1.63	*
K5-2	2.03	1.51	1.50	1.56	*
K6-0	0.25	0.29	1.21	1.56	0.88
K6-1	1.95	2.01	0.58	0.56	0.36
K6-2	1.74	1.58	0.83	0.93	0.74
K7-0	0.96	0.31	1.71	1.54	1.02
K7-1	0.40	1.34	1.19	0.40	1.90
K7-2	0.73	1.27	1.55	0.78	1.45
K8-0	0.29	1.25	0.07	0.98	1.09
K8-1	2.17	2.04	1.96	1.74	0.08
K8-2	1.83	1.84	1.67	1.58	1.81
K9-0	1.39	0.20	0.22	0.24	0.26
K9-1	0.38	1.08	0.39	1.79	0.69
K9-2	0.70	1.80	0.68	1.71	1.71

* Mice that died from other causes than scrapie

Table S2: Survival time (individual values) of RML-infected C57/Bl6 mice immunised with virus-like particles.

	**Incubation time [days]**								
**Construct**	Animal 1	Animal 2	Animal 3	Animal 4	Animal 5	**mean value**	standard deviation	**elongation [d] to untreated control**	**log rank test**
**control**	209	197	200	200	202	**201.6**	4.5	**-**	-
**VP1/2 control**	203	211	218	223	*	**213.75**	8.7	**12.15**	0.017
**Construct K1**	232	204	281	197	267	**236.2**	37.2	**34.6**	n.s.
**Construct K2**	232	207	232	211	200	**216.4**	14.8	**14.8**	n.s.
**Construct K3**	207	209	134	210	207	**193.4**	33.2	**-8.2**	n.s.
**Construct K4**	210	223	162	211	218	**204.8**	24.5	**3.2**	n.s.
**Construct K5**	223	200	223	225	*	**217.75**	*11.9*	**16.2**	n.s.
**Construct K6**	232	210	197	202	209	**210**	*13.4*	**8.4**	n.s.
**Construct K7**	232	218	218	211	225	**220.8**	8.0	**19.2**	n.s.
**Construct K8**	204	210	217	211	217	**211.8**	5.4	**10.2**	n.s.
**Construct K9**	237	249	249	218	249	**240.4**	13.6	**38.8**	p≤0.015

* Mice that died from other causes than scrapie

Figure S1

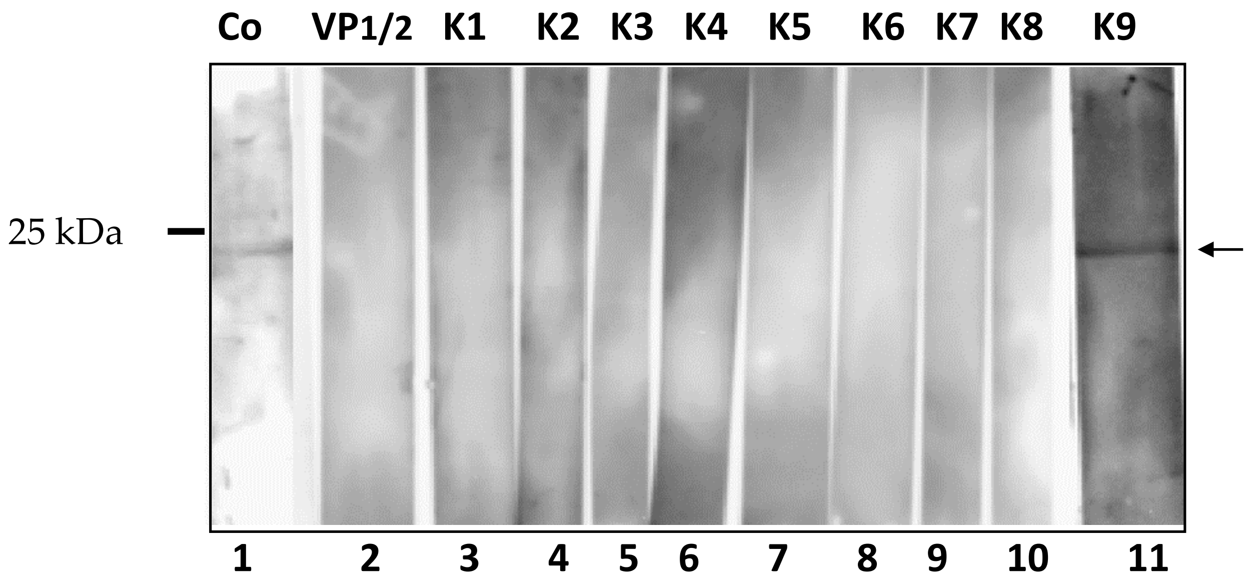

Figure S1. Western blot analysis of pooled sera from animals immunized with VP1/2 control (VP1, lane 2), K1 (lane 3), K2 (lane 4), K3 (lane 5), K4 (Lane 6), K5 (lane 7), K6 (lane 8), K7 (lane 9), K8 (lane 10), K9 (lane 11). Each lane contains blotted recombinant murine prion protein separated on an 16% SDS-PAGE and probed with corresponding serum pools. mAb SAF70 (lane 1) was used as a positive control (Co).

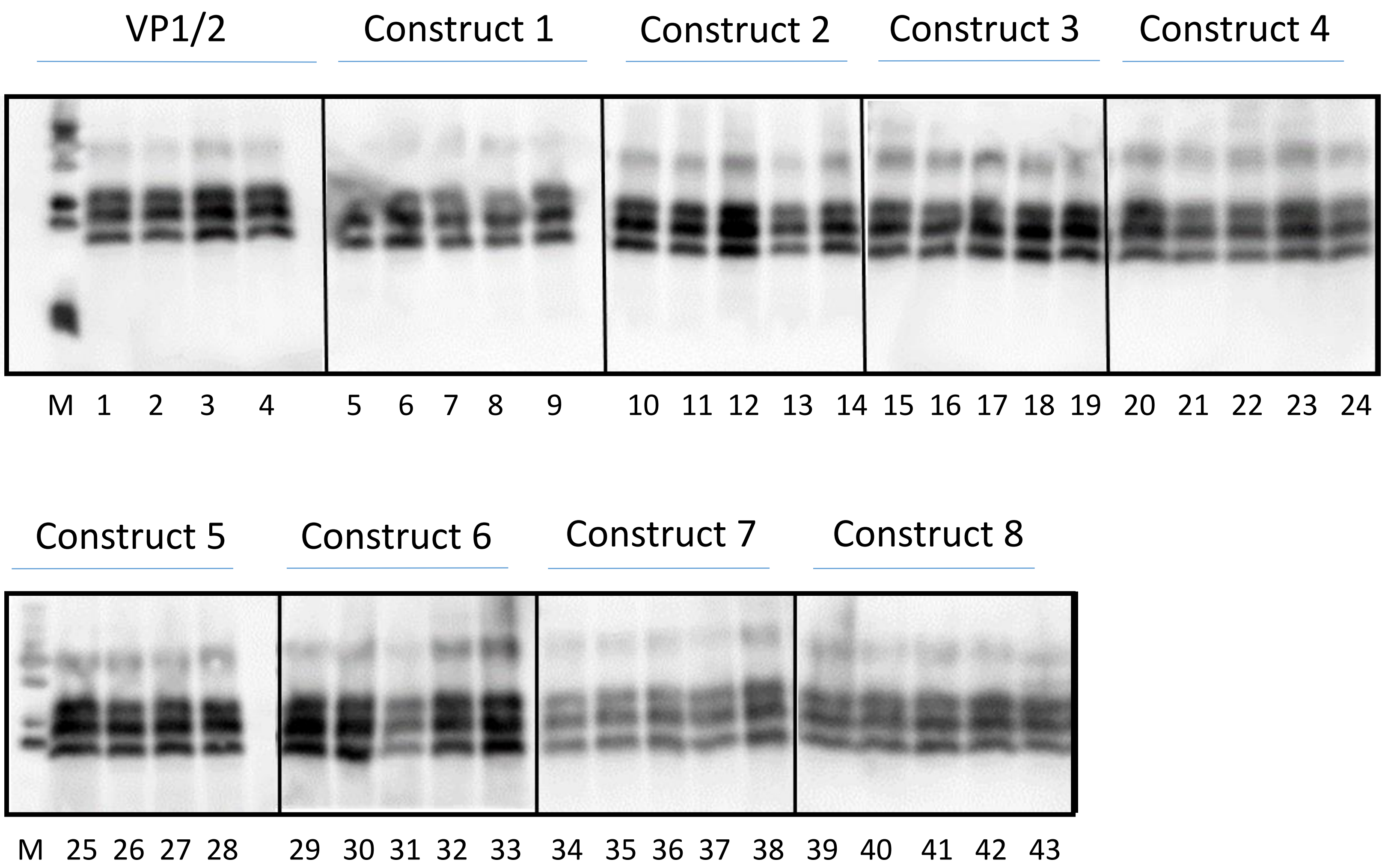

Figure S2. PrP^{Sc} accumulation in the brains of selected mice challenged with RML. Immunoblot analysis of RML-infected mouse brain homogenate digested with proteinase K (PK). PrP^{Sc} of scrapie infected mice after VP1/2 immunisation (lane 1-4), after immunisation with construct 1 (lanes 5-9), with construct 2 (lanes 10-14), with construct 3 (lanes 15-19), with construct 4 (lanes 20-24), with construct 5 (lanes 25-28), with construct 6 (lanes 29-33), with construct 7 (lanes 34-38) and with construct 8 (lanes 39-43) Detection of PrP^{Sc} was carried out with mAb SAF70. (M) molecular weight marker.
